# Placental transfusion: a review

**DOI:** 10.1038/jp.2016.151

**Published:** 2016-09-22

**Authors:** A C Katheria, S Lakshminrusimha, H Rabe, R McAdams, J S Mercer

**Affiliations:** 1 Division of Neonatology, Neonatal Research Institute, Sharp Mary Birch Hospital for Women & Newborns, San Diego, CA, USA; 2 Department of Pediatrics (Neonatology), University at Buffalo, Buffalo, NY, USA; 3 Academic Department of Pediatrics, Brighton and Sussex Medical School, Brighton, UK; 4 Department of Pediatrics, University of Washington, Seattle, WA, USA; 5 Division of Midwifery, University of Rhode Island, Kingston, RI, USA; 6 Division of Midwifery, Alpert School of Medicine, Brown University, Providence, RI, USA; 7 Division of Midwifery, Women and Infants Hospital of Rhode Island, Providence, RI, USA

## Abstract

Recently there have been a number of studies and presentations on the importance of providing a placental transfusion to the newborn. Early cord clamping is an avoidable, unphysiologic intervention that prevents the natural process of placental transfusion. However, placental transfusion, although simple in concept, is affected by multiple factors, is not always straightforward to implement, and can be performed using different methods, making this basic procedure important to discuss. Here, we review three placental transfusion techniques: delayed cord clamping, intact umbilical cord milking and cut-umbilical cord milking, and the evidence in term and preterm newborns supporting this practice. We will also review several factors that influence placental transfusion, and discuss perceived risks versus benefits of this procedure. Finally, we will provide key straightforward concepts and implementation strategies to ensure that placental-to-newborn transfusion can become routine practice at any institution.

## Introductions

One essential goal of neonatal critical care is to deliver adequate oxygen to meet tissue demand. Increasing fetal hemoglobin (Hb) by placental transfusion is an extremely effective method of enhancing arterial oxygen content, increasing cardiac output and improving oxygen delivery. Placental transfusion is the transfer of residual placental blood to the baby during the first few minutes of age, and can be accomplished by three different methods: delayed cord clamping (DCC), intact umbilical cord milking (I-UCM), and cut-umbilical cord milking (C-UCM). The goal of placental transfusion is to facilitate transfer of blood volume from the placenta to the newborn. Fetal blood circulates in the feto-placental unit throughout gestation. Owing to the relatively large size of placenta compared with the fetus at mid-term, blood is equally distributed between the fetus and placenta. By term gestation, about one-third of the blood flows through the placenta and two-thirds flows through the fetus at any point in time.^1^ Immediate cord clamping (ICC) results in ~30% of feto-placental blood volume remaining in the placenta, whereas DCC reduces residual placental blood to 20% of the feto-placental blood volume by 60 s and to 13% by ~3–5 min.^2^

## Assessment of the magnitude of placental transfusion

Different methods are utilized to calculate the magnitude of placental transfusion in clinical studies. Extending the time before cord clamping leads to a gradual increase in fetal-neonatal blood volume with a corresponding decrease in residual placental blood volume.^2^ Residual placental volume can be measured by placing the cut end of the umbilical cord (and placenta after expulsion) in a funnel or by collecting placental blood with a dedicated kit.^3^ Neonatal red blood cell volume or whole-blood volume has been calculated using radio-labeled chromium or biotin.^4, 5^ Both calculation methods require mixing the infant's blood with a tracer substance and drawing blood after a brief waiting period, which makes this an undesirable approach in healthy infants. Measurement of Hb or hematocrit during the first 24 h can provide a crude assessment of placental transfusion, but is dependent on fluid shift from the intravascular compartment. Serum ferritin levels during infancy assess iron load secondary to placental transfusion and are commonly used in long-term studies, although inflammation elevates this acute phase reactant, which can hinder interpretation of values. Measuring the change in body weight at birth and at the conclusion of placental transfusion is a non-invasive approach to assess blood volume increases, but may be less accurate that more direct methods.^6, 7^ The exact method of assessing magnitude of transfusion must be taken into consideration when comparing different studies evaluating placental transfusion.

## Factors determining placental transfusion

**Figure 1:**
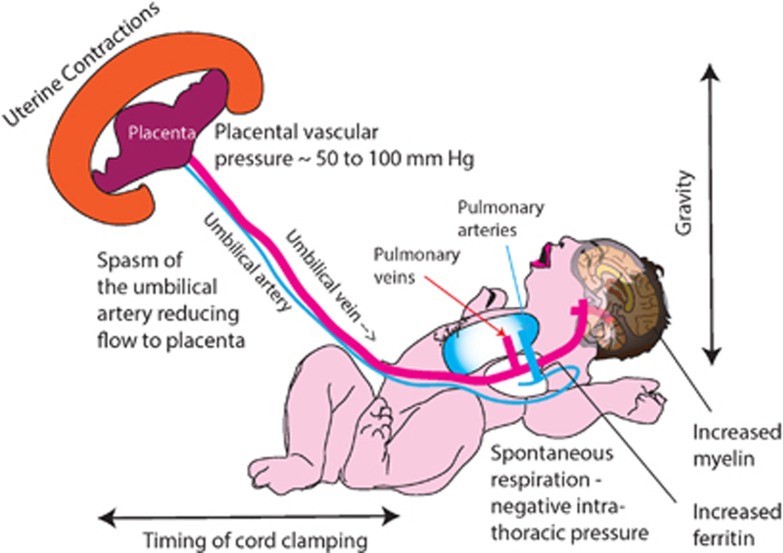
Factors influencing placental transfusion with delayed cord clamping (DCC). Timing of cord clamping, uterine contractions, reduced neonate-to-placental flow due to umbilical arterial spasm, spontaneous respirations and gravity influence the magnitude of transfusion. Reported long-term benefits are shown.

Several factors including cord clamping time, uterine contractions, umbilical blood flow, respirations and gravity have an important role in determining placental transfusion volumes (Figure 1).

### Time of cord clamping

Farrar *et al.*^6^ estimated placental transfusion for both vaginal and cesarean births by measuring infant weight gain in the first 5 min after birth with their cords left intact. The mean amount of placental transfusion was 81 ml (range, 50–163 ml) or 25 ml kg^−1^ (range, 16–45 ml kg^−1^). The authors estimated that placental transfusion contributed to about 20% of the infant's blood volume at birth. This estimate is supported by a classical work of Yao,^2^ which demonstrated that a term infant's blood volume is approximately 70 ml kg^−1^ following ICC compared with ~90 ml kg^−1^ with DCC (3 min). Aladangady *et al.*^4^ showed that brief DCC (∼30–45 s) in preterm infants resulted in an 8–24% increase in blood volume (2–16 ml kg^−1^ at cesarean birth and 10–28 ml kg^−1^ at vaginal birth). In term and preterm births, DCC results in more blood being transferred to the infant and is proportional to the time delayed.^2^

### Uterine contractions

Uterine contractions are the primary determinant of placental transfusion in spontaneous deliveries with DCC. The initial uterine contraction that expels the fetus contributes to 25–30% of placental transfusion. The intrauterine umbilical venous pressure is high (~40–50 mm Hg in between contractions and increasing to 100 mm Hg during contractions) and provides a gradient for blood flow from the placenta to the neonatal right atrium. In between contractions, the umbilical venous flow is approximately equal to umbilical arterial flow with minimal net transfer of blood to the fetus. Uterine contractions during the third stage of labor significantly increase the placenta-to-neonatal right atrial gradient and may facilitate 50% of placental transfusion.^8^

### Umbilical blood flow

During fetal life ~29% of the combined ventricular output (equivalent to 130 ml kg^−1^ fetal body weight) flows through the umbilical arteries to the placenta and returns to the fetus via the umbilical vein. After birth, during the third stage of labor, the umbilical arteries constrict, often within 45 s, minimizing blood flow from the neonate to the placenta, whereas the umbilical vein remains patent facilitating placental transfusion.^9^ The onset of respirations and increase in neonatal PaO_2_ may facilitate umbilical arterial constriction.

### Spontaneous breathing and respirations

Spontaneous breathing and crying creates negative intrathoracic pressure and increases the gradient between placental vasculature and fetal right atrium facilitating placental transfusion. Boere *et al.*^10^ observed intermittent flow every 1.5 s in the umbilical vein by Doppler of the umbilical cord possibly reflecting a respiratory rate of 40 per min. However, in the presence of strong uterine contractions (with pressure gradients of ~100 mm Hg), respiration does not appear to further enhance placental transfusion.^11^ Following cesarean section with absent uterine contractions, spontaneous respiration might have a more important role in facilitating placental transfusion. Philip *et al.*^12^ evaluated 29 full-term babies delivered by elective cesarean section. Residual placental blood volume and change in hematocrit were measured. Increasing duration of respiration resulted in increasing amounts of placental transfusion. Positive pressure ventilation (PPV) increases intrathoracic pressure. PPV increases pulmonary blood flow and reduces pulmonary vascular resistance, but its effect on placental transfusion is not clear. Creasy *et al.* ventilated lambs with PPV, whereas on placental circulation and compared with lambs where the cord was cut before PPV. No difference in blood volume was observed after 5 min of ventilation.^13^ Unlike spontaneous respiration, PPV does not appear to enhance placental transfusion.

Recent animal and human studies suggest that early cord clamping before the onset of spontaneous respirations appears to adversely affect cerebral perfusion during fetal to neonatal transition, likely due to the increased afterload seen by the left ventricle and the decrease preload provided by umbilical venous return.^14, 15, 16^ Lung aeration triggers an increase in pulmonary blood flow, which supplies most of the preload to the left ventricle; if cord clamping precedes onset of respirations, ventricular preload falls, decreasing left ventricular output thereby decreasing carotid artery blood flow and cerebral blood flow. Hence, hemodynamic stabilization after delivery, which maintains cerebral blood flow, is a potential strategy to preserve germinal matrix vasculature and reduce severe intranventricular hemorrhage (IVH). It seems logical that a sufficient placental transfusion by either DCC or UCM, would augment cerebral blood flow at birth and reduce IVH, which has been demonstrated by a study using Near InfraRed Spectroscopy,^17^ and has been supported by several meta-analyses.^18, 19^ Ersdall *et al.*^16^ demonstrated that in term infants without respirations before cord clamping had an increased risk of death or NICU admission.

**Figure 2:**
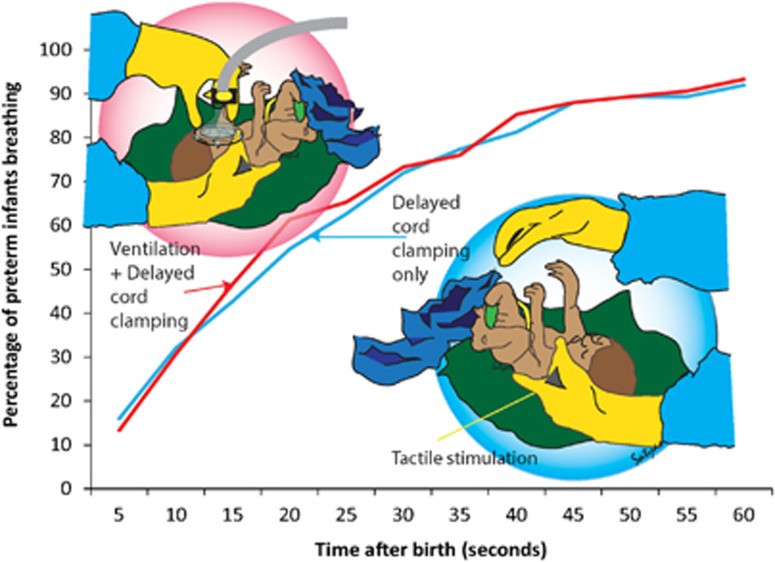
Onset of spontaneous breaths in preterm infants following DCC with stimulation (blue line) and positive pressure ventilation (PPV) with DCC (red line).

In premature newborns, it remains unclear if successful placental-to-newborn transfusion requires a few gasping breaths or PPV. A recently completed enrollment in a feasibility trial in which preterm newborns were randomized to receive DCC with stimulation or ventilation (VDCC) demonstrated no difference between the provision of early continuous positive airway pressure, PPV or gentle tactile stimulation (rubbing the back) during DCC of 60 s.^20^ The mean time to establish breathing was similar in each arm (about 25 s), with over 90% of infants establishing breathing before cord clamping by 60 s (Figure 2). However, infants receiving DCC had a greater duration of stimulation than VDCC. This suggests that although ventilation during DCC is feasible, it is not superior to DCC alone when infants receive adequate stimulation. Caretakers should consider providing adequate stimulation before cord clamping.

We are unaware of any current studies investigating resuscitation of term infants at risk for asphyxia with an intact umbilical cord. However, compared with early cord clamping, DCC has recently been shown to increase myelin content at 4 month of age in brain regions associated with motor, visual and sensory processing/function compared with early cord clamping, suggesting DCC may be a potential neuroprotective treatment.^21^ Studies using autologous umbilical cord blood (mononuclear cells) to treat hypoxic ischemic encephalopathy demonstrate reduced markers of brain damage, neuroinflammation and neural apoptosis.^22^ These autologous stem cells are neuroprotective if administered within 12 h after perinatal asphyxia, are well tolerated, and offer a feasible treatment for infants following hypoxic ischemic encephalopathy. Resuscitation with an intact umbilical cord should offer the infant their full allotment of red blood cells, blood volume and the whole array of stem cells available in cord blood. Obtaining cord gases should not preclude DCC. Reliable umbilical cord gases can be obtained from an intact cord, preventing the need to cut the cord to obtain cord blood gases.^23^

### Effects of gravity: positioning of the infant in relation to placenta

Yao *et al.*^2^ found that gravity affects the amount of placental transfusion. Holding the neonate high above the placenta (head 40–60 cm above) decreases placental transfusion similar to ICC.^24^ A recent study found no difference in infant weights after DCC for 2-min with infants placed on the maternal abdomen versus at the introitus.^7^ However, total weight gain was half of what was previously found^25, 26^ indicating that 2 min may not be enough time for a full placental transfusion for the term infant.^7^ Mercer *et al.*^27^ found that term infants placed on the maternal abdomen immediately after birth who were assigned to DCC for 5-min received a significantly larger placental transfusion than those with a two-minute delay.

## Cord milking technique and terminology

Currently, in the literature, the term umbilical cord milking has been used interchangeably with milking the cord when it is intact (connected to the placenta) and after it is cut and separated from the placenta. We propose the terms intact cord milking and cut cord milking as described below.

### Intact umbilical cord milking

**Figure 3:**
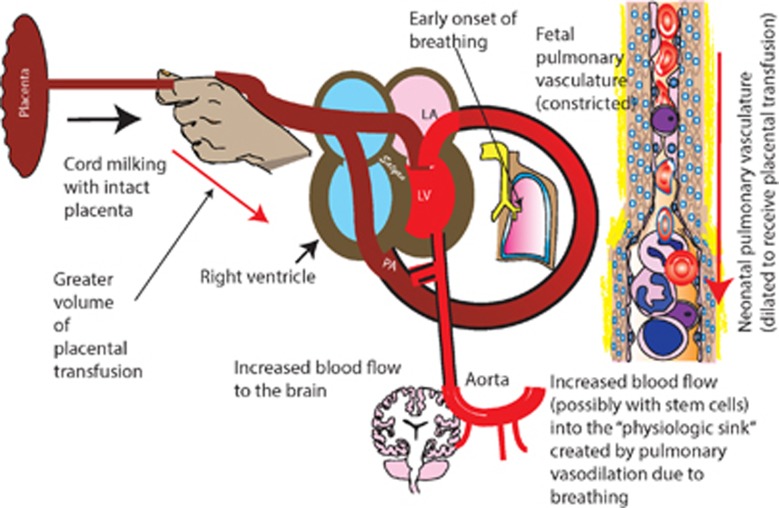
Placental transfusion through cord milking with an intact cord (I-UCM) attached to the placenta. Placental blood can potentially increase blood flow to the brain and lungs. Pulmonary vasodilation in response to spontaneous respiration or crying can create a ‘physiologic sink' to accommodate placental blood. Placental blood is a rich source of fetal red blood cells and stem cells.

An alternative to DCC is I-UCM where the unclamped umbilical cord is grasped and blood is pushed (‘stripped') toward the infant two to four times before it is clamped. This procedure transfers blood into the preterm neonate and can be performed within 20 s.^28^ Careful attention should be paid to how the cord milking is performed, for example, how many times and whether the cord is kept intact. Cord milking before clamping improves the pulmonary blood flow immediately at birth and assists lung expansion at the onset of respirations (Figure 3). This may explain why milking may assist with earlier onset of breathing compared with DCC. In a pilot study comparing 60 s of DCC with 4 times I-UCM of the intact cord, more infants breathed before cord clamping with I-UCM compared with DCC (74 versus 53%).^28^ Jaykka *et al.*^29^ demonstrated that the alveolar patency occurs in response to the filling of the surrounding capillaries. Recording of electrocardiographic changes associated with cord milking-induced increases in lung blood demonstrate longer P wave, PR and QT_C_ intervals in newborn with cord milking compared with those who had ICC.^30^

A recent meta-analyses of seven randomized-controlled I-UCM trials in premature newborns delivered at <33 weeks demonstrated that neonates who undergo I-UCM have higher Hb, lower risk for chronic lung disease and decreased IVH of all grades compared with those who undergo ICC. Cord milking may offer an advantage over DCC in newborns who are deemed too unstable to wait for DCC and who are at the highest risk of severe IVH and death.^31, 32, 33^ Current published guidelines regarding the management at delivery of premature newborns only recommend DCC if it is ‘feasible,' ‘possible' or ‘if the infant does not require resuscitation.' I-UCM has been shown to deliver a greater placental transfusion than DCC in premature newborns delivered by cesarean section.^28^ I-UCM can also be performed in any low resource setting and provides adequate placental transfusion to the premature newborn without delay, making it feasible for depressed infants as well.

We recommend milking the umbilical cord four times for preterm infants^28, 34^ and five times^35^ for term infants. The infant is usually held in a neutral position relative to the placenta. A second person may be helpful, particularly during vaginal delivery to hold and dry the infant during the procedure.

The hemodynamic changes in cerebral and pulmonary circulation following cord milking are still being investigated. Unpublished data from Lakshminrusimha's laboratory demonstrates increased cerebral and pulmonary blood flow with cord milking in preterm lambs (Figure 3). Previous work in humans has demonstrated increased heart rate and oxygen saturation within the first 5-min of birth with I-UCM compared with ICC.^36^ Although repeat cord milking allows some back flow of blood towards the placenta via the umbilical arteries, it allows the afterload of the left ventricle to remain low, whereas blood is being infused into the pulmonary circulation after each milking. The majority of cord milking trials in term and preterm deliveries employed milking before cord clamping and have demonstrated benefits in blood pressure, IVH, chronic lung disease and death without any reported complications. Clinical studies have not demonstrated any negative effects on neurological outcomes (including IVH) in preterm infants following cord milking.^37^

### Cut-umbilical cord milking

**Figure 4:**
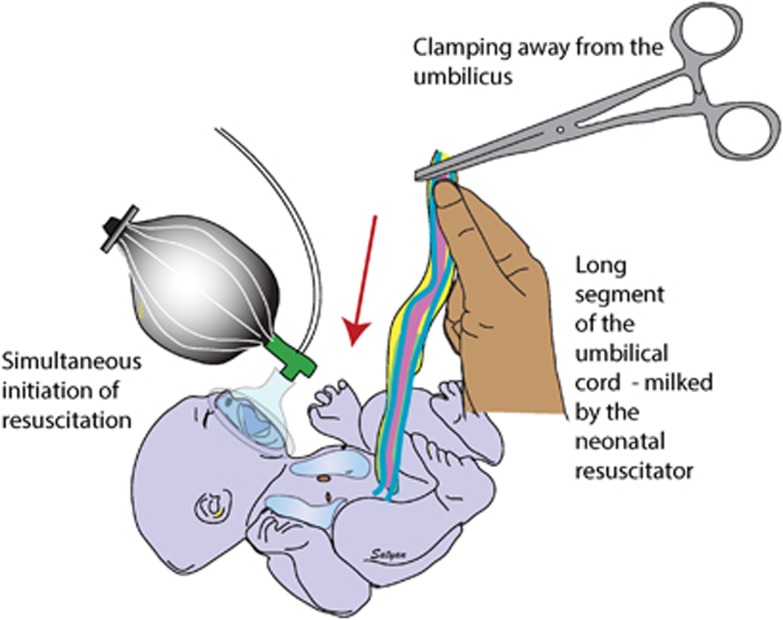
Cut-umbilical cord milking (C-UCM) is performed by clamping away from the fetus and retaining a long segment of the umbilical cord that can be milked by the neonatal provider simultaneously with resuscitation.

Another technique, used more often in Asia, involves clamping and cutting a long segment of the umbilical cord immediately at birth and passing the baby and the long cord to the pediatric provider, called C-UCM (Figure 4).^38, 39^ The pediatric provider then untwists the cord and milks the entire contents into the baby. Milking the cord 2–3 times before clamping may produce a similar placental transfusion as C-UCM.^38^ So far, there are no prospective trials comparing the two methods of cord milking. Although the physiological rationale may be problematic in light of recent animal and human reports of adverse outcomes of clamping before the onset of respiration,^14, 16^ improved neurodevelopemental outcomes have been recently reported in premature infants compared with ICC.^39^

## Risks and benefits of DCC and cord milking

Theoretical risks from placental transfusion often mentioned include over-transfusion, symptomatic polycythemia, jaundice, hypothermia, persistent pulmonary hypertension and delayed resuscitation. However, none of these risks have appeared in the current randomized-controlled trials and meta-analyses on term or preterm infants.

In considering over-transfusion, one needs to understand that to achieve an adequate transfer of gases necessary for breathing, the infant needs ~50% of his cardiac output to flow through the newborn lung instead of the ~10% that the fetal lung received *in utero*. This is accomplished by the large volume of blood moving from the fetal ‘lung' (placenta) to newborn lung. Thus, placental transfusion rapidly creates an increase in the circulatory bed in the lung. There is no evidence of over-transfusion in any recent randomized controlled trials.^41, 42^ Along with increased blood volume in the lung, the placental blood is also distributed to the peripheral circulation creating better perfusion and less hypothermia.^43, 44^ No hypothermia has been reported in any of the meta-analyses involving term or preterm infants.^19, 26^

Concern has been raised as to whether preterm infant hearts can handle this full physiologic blood volume. *In utero*, the fetal heart is pumping about 110 ml kg^−1^ through the body, out through the cord to the placenta and back to the body.^9^ After birth, with a full placental transfusion, the infant's heart is pumping about 90 ml kg^−1^ only within his body. Backes compared ICC and DCC in term infants with critical congenital heart defects and found that infants with DCC required fewer transfusions after surgery and displayed no adverse effects.^45^

A widely held belief is that there is a link between DCC, symptomatic polycythemia and hyperbilirubinemia. Although concern has been raised that DCC may cause infants to get too many RBCs, the red cells and blood volume expand together, increasing capillary beds throughout the body^43^ and providing more iron.^9, 43^ There are no reports of symptomatic polycythemia in either recent meta-analyses.^42^

Hyperviscosity and its effect on pulmonary vascular resistance following placental transfusion have led to concerns about the increased risk of persistent pulmonary hypertension of the newborn (PPHN). Studies have suggested that pulmonary arterial pressure is elevated following placental transfusion.^46^ We speculate that placental transfusion at the onset of breathing elicits an ‘erectile' response and distends the pulmonary vasculature leading to increased pulmonary arterial pressure and is associated with increased pulmonary blood flow. In fact, infants with PPHN tend to have lower Hb compared with infants with respiratory distress without PPHN suggesting that placental transfusion may be protective against pulmonary hypertension by promoting pulmonary blood flow and increasing oxygen carrying capacity.^47^ It has also been suggested that high pulmonary arterial pressure may be protective against fluctuating cerebral blood flow by reducing ductal shunt in extremely preterm infants.^48^ We speculate that improved cerebral blood flow, oxygen carrying capacity, increased pulmonary arterial pressure and reduced ductal shunt following placental transfusion may contribute to reduced IVH observed in preterm infants following DCC.^19^

In eight studies involving over 1000 neonates, there was no significant difference in risk of jaundice within 24–48 h.^41^ In the most recent meta-analysis of 1828 infants in five studies, there were no significant differences in clinical jaundice.^49^ Although a slight increase (2%) in the need for phototherapy with DCC was reported, this must be weighed against recent evidence that higher levels of bilirubin within normal limits may offer neuroprotection to the infant.^50^ Even using DCC in infants with alloimmunization requiring intrauterine transfusion, Garabedian *et al.*^51^ found no increase in jaundice. It Is important that pediatric providers be blinded to the randomization of infants in studies examining jaundice and polycythemia as beliefs are widespread and do influence practice.^52^

There are no reports of differences in Apgar scores or low temperature on admission in studies of term or preterm infants with ICC versus DCC. Improved heart rate and spO_2_ have been shown with UCM compared with ICC.^36^ The additional blood volume provided during DCC or UCM may be advantageous during resuscitation.

There is still a need for pragmatic, well-designed trials comparing placental transfusion techniques with meaningful clinical endpoints, such as long-term neurodevelopmental follow-up. There are only a few small studies that have published long-term folllowup. Mercer *et al.*^53^ demonstrated improved motor function at 18–22 month corrected age with DCC (*n*=161) combined with a one-time UCM compared with ICC. Rabe *et al.*^37^ demonstrated similar outcomes with UCM compared with DCC at 2 (*N*=39) and 3.5 (*N*=29) years of age. Lastly, Andersson *et al.*^54^ demonstrated improved fine motor and social domain scores at 4 years of age, especially in boys following DCC. Although all of these trials are reassuring they were not adequately powered for neurodevelopmental follow-up. There is at least one large multicenter trial (Australian placental transfusion study trial, *N*=1600) comparing DCC to ICC that is adequately powered for neurodevelopmental outcomes. To fully explore and understand the long-term impact of DCC and UCM, future research must be adequately powered and included neurodevelopmental follow-up.^55^

## Recommendations for term and preterm infants

For preterm infants, current studies report that DCC of 60–120 s results in significant benefits. All of the preterm studies hold the infant lower than placenta to facilitate rapid transfusion. No studies have used a protocol of placing the preterm infant on the maternal abdomen. At cesarean section the infant is usually placed on the mother's thighs or held down to side. If cord is still full at the end of the time allotment, one can milk the cord to obtain maximal transfusion of fetal blood to the infant. To prevent hypothermia, one can wrap the infant in a warm sterile blanket and provide gentle stimulation or place the preterm newborn in plastic wrap or bag.

For term infants, DCC for at least 3 min provides a full placental transfusion when held at the level of the perineum.^2^ In stable neonates, if placed on the mother's abdomen, delaying clamping the cord past 3 min should be considered as it may take two to three maternal contractions for the full placental transfusion to occur.^27, 35^ One needs to remember that there is no rush to cut the cord in these infants. One can ‘Wait for White' or until the cord is flat and white indicating that the infant has received most of the blood left in the placenta. It is important to avoid pressure or traction on cord as this is believed to cause the vessels to spasm. *In situations* where the infants is depressed or during cesarean section milking the cord several times produces a similar placental transfusion without delaying resuscitation or time on the operative field.

## Implementing a placental-to-newborn transfusion protocol

The safety and beneficial effects of placental-to-newborn transfusion through the practice of DCC or UCM have been consistently demonstrated in multiple clinical trials.^19, 49, 56^ Despite this evidence-based practice (EBP) favoring placental-to-newborn transfusion strategies over ICC at birth, implementation of DCC and/or UCM to improve outcomes and/or reduce risks in newborns has not been widespread.^57, 58^ This discordancy between usual care practices and EBP is not uncommon in healthcare, a setting where providers with a spectrum of education, training, beliefs and personality are often cautious to adopt new practices.^59^ Implementing a new clinical practice or significantly modifying a pre-existing practice can be a challenging task that often requires surmounting existing barriers, both obvious and obscure, such as colleagues resistant to change and differences in cultural and social beliefs/attitudes. To successfully accomplish this challenging task, individuals or a group of leaders interested in implementing a placental transfusion strategy need to have a committed and patient approach to overcome inertia common to established medical practices, such as ICC.^60^ Poor implementation may result in an unfavorable assessment of EBP effectiveness, when in actuality, a weak implementation process, not the EBP, is the cause of the inferior outcome.^61^

**Figure 5:**
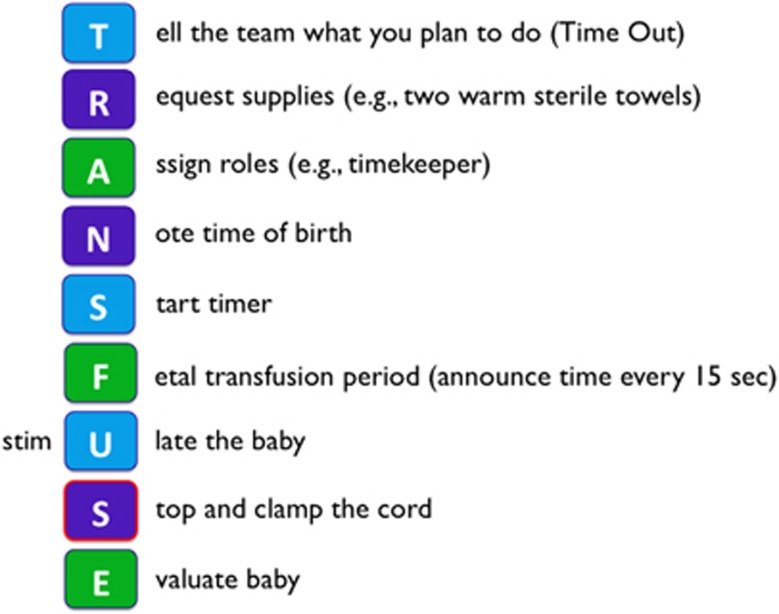
A simple placental transfusion protocol that can be posted in the delivery room.

Implementation of a placental transfusion protocol is an adaptive endeavor that requires a supportive environment to promote an effective, consistent and sustainable process that can include a variety of key elements. An essential starting point in this process is a core group of leaders, working as a cohesive team, to help to champion a change (for example, DCC and/or UCM) in a well-established clinical practice such at ICC. To properly inform strategy development for making DCC or UCM operational, these leaders need to determine if their organization is ready for the practice change by assessing existing logistical and operational factors pertaining to newborn deliveries. The core group of leaders will need to recruit and obtain buy-in from other stakeholders (for example, neonatologists, pediatricians, obstetricians, midwives, neonatal and obstetrical nurses, respiratory therapists, neonatology fellows, and pediatric and obstetrical residents) that will be affected by the new protocol. A variety of educational methods may be utilized to disseminate knowledge to potential stakeholders (for example, grand rounds, discussions at noon conferences or division meetings, teaching during resident orientation, simulation exercises and online training modules). Teamwork should be emphasized with ongoing input sought from stakeholders to promote constructive feedback, address concerns, and endorse safe, efficient, and effective practice. An easy to follow placental transfusion protocol can be developed that can be posted in the delivery rooms to remind staff of the important steps to perform DCC or UCM (Figure 5). As compliance to a new protocol may diminish over time,^60^ methods to foster compliance (for example, delivery pre-briefs, checklists, mandatory data field in the electronic medical record) and measure compliance (for example, quality improvement project conducted by a stakeholder) should be considered. Encouraging stakeholders on their important roles in carrying out an EBP may strengthen working relationships and improve practice compliance, which will help achieve the original outcome goals. Qualitative research with parents whose babies were enrolled in placental transfusion trials revealed a positive attitude but also the request for early information during the pregnancy.^62^ The latter would provide women with the chance to write placental transfusion into their birth plans.

Depending on an institution's placental transfusion guidelines, protocol compliance may appear satisfactory, whereas the actual practice of DCC may be low if the protocol guidelines are too loose and allow for easy exclusion of newborns based on policy clauses. Jelin *et al.*^63^ found that despite numerous methods of policy dissemination, compliance with their cord clamping policy was 88%, but DCC only occurred in 49%. Theoretical concerns about hyperbilirubinemia and delayed resuscitation resulted in a liberal exclusion clause within their policy. This practice provision lead to increased policy compliance, but allowed more newborns to be disqualified, thus decreasing the number of infants who actually received DCC. Organizations practicing DCC and/or UCM should carefully consider which criteria should be grounds for early cord clamping.

## Conclusion

ICC is a practice that disrupts the normal physiologic processes that occur at birth. ICC was adopted as a matter of convenience without any study or evidence regarding its impact on infants. Placental transfusion, whether by DCC or UCM, has been shown to reduce IVH, the need for transfusions and inotropes, and necrotizing enterocolitis in preterm infants and improve iron stores in the first 6 months of age for term infants. Placental transfusion should be considered at every delivery as it can have a marked impact on the outcomes of newborns. Centers currently performing ICC should review the evidence for placental transfusion with key stakeholders and adopt a team-based approach to ensure that this technique is eliminated from routine practice.
